# P-596. Immunobridging Demonstrating Effectiveness of the Bivalent Respiratory Syncytial Virus (RSV) Prefusion F Subunit Vaccine in Adults 18-59 Years of Age at High Risk of Severe RSV Disease in a Phase 3 Trial: The C3671023 MONeT Study Results

**DOI:** 10.1093/ofid/ofae631.794

**Published:** 2025-01-29

**Authors:** Matthew Davis, William Towner, Elliot N DeHaan, Qin Jiang, Wen Li, Farah Rahman, Michael Patton, Hayley Wyper, Maria Maddalena Lino, Uzma N Sarwar, Zaynah Majid-Mahomed, Saumil Mehta, William Howitt, Kevin Cannon, Elena Kalinina, David Cooper, Kena A Swanson, Annaliesa S Anderson, Alejandra C Gurtman, Iona Munjal

**Affiliations:** Rochester Clinical Research, Rochester, New York; Kaiser Permanente Southern California, Pasadena, California; Pfizer, Pearly River, New York; Pfizer, Pearly River, New York; Pfizer Vaccine Clinical Research, Collegeville, Pennsylvania; Pfizer, Pearly River, New York; Pfizer, Vaccine Research and Development, Hurley, England, United Kingdom; Pfizer, Inc. Vaccine Research & Development, Marlow, England, United Kingdom; Pfizer, Pearly River, New York; Pfizer, Pearly River, New York; Pfizer, Pearly River, New York; AIM Trials, Plano, Texas; QPS Missouri, Springfield, Missouri; Accellacare, Wilmington, North Carolina; Pfizer, Pearly River, New York; Pfizer, Pearly River, New York; Pfizer, Pearly River, New York; Pfizer, Pearly River, New York; Pfizer, Pearly River, New York; Pfizer Inc

## Abstract

**Background:**

C3671023 Substudy A (NCT05842967) is a phase 3, randomized, double-blinded, placebo-controlled study to assess the safety and immunogenicity of Pfizer’s RSVpreF in adults 18-59 years of age at high risk of severe RSV disease due to chronic medical conditions, including pulmonary, cardiovascular, hepatic, renal, and metabolic (including diabetes mellitus) disorders. Primary objectives were to assess safety and to demonstrate a non-inferior immune response in adults 18-59 years of age at high risk of severe RSV disease compared to adults age ≥ 60 from the pivotal phase 3 C3671013 study which demonstrated efficacy against RSV Lower Respiratory Tract Illness (LRTI).

**Table 1:**
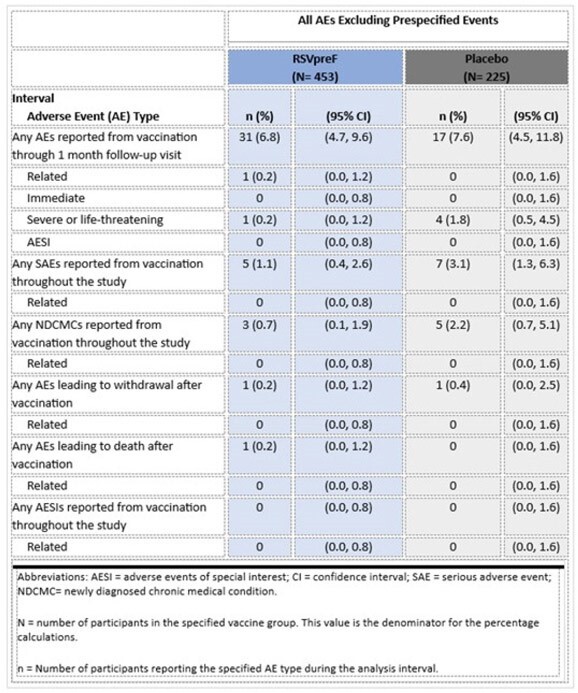
Number of Participants Reporting at Least 1 Adverse Event (Excluding Prespecified Events) for Each Analysis Interval After Vaccination with RSVpreF

**Methods:**

Participants were randomized 2:1 to receive 1 dose of RSVpreF or placebo (453 RSVpreF: 225 placebo). Sera collected before and at 1 month after vaccination were tested concurrently with sera collected from ∼400 RSVpreF recipients from the C3671013 study. Reactogenicity events were collected for 7 days following vaccination. Adverse events (AEs) were collected through 1 month following vaccination; adverse events of special interest (AESIs), newly diagnosed chronic medical conditions (NDCMCs), and serious AEs were collected throughout the study. Non-inferiority (NI) would be declared if the lower bounds of the 95% confidence interval (CI) of adjusted Geometric Mean Ratio (GMR) of neutralizing titers (NTs) were > 0.667 and lower bounds of the 95% CI of seroresponse rate differences were > -10% for RSV A and RSV B.

**Table 2:**
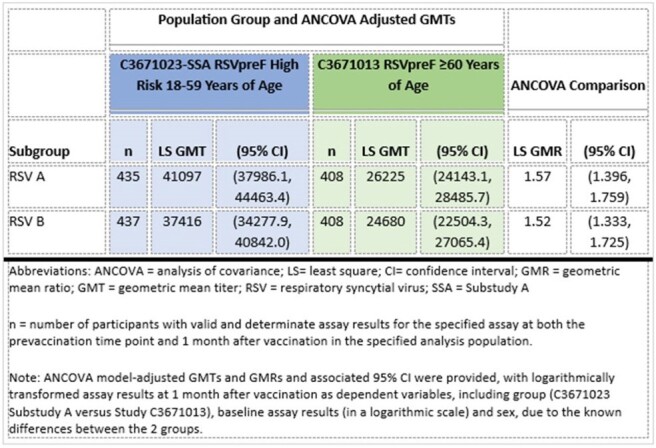
Comparison of Model-Adjusted RSV Neutralizing - GMTs at 1 Month After Vaccination with RSVpreF- 18 Through 59 Years at High Risk (C3671023) versus 60 Years and Older (C3671013)

**Results:**

Proportions of participants with local reactions were higher in the RSVpreF group; systemic events were generally similar in both groups (Figure 1). Proportions of participants reporting any AEs were similar between RSVpreF and placebo through 1 month following vaccination and throughout the study (Table 1).

RSV A and RSV B NTs 1 month after RSVpreF vaccination in adults 18-59 years of age at high risk were non-inferior to adults ≥ 60 years of age (Table 2). NI was met for seroresponse rates (Table 3).

**Table 3:**
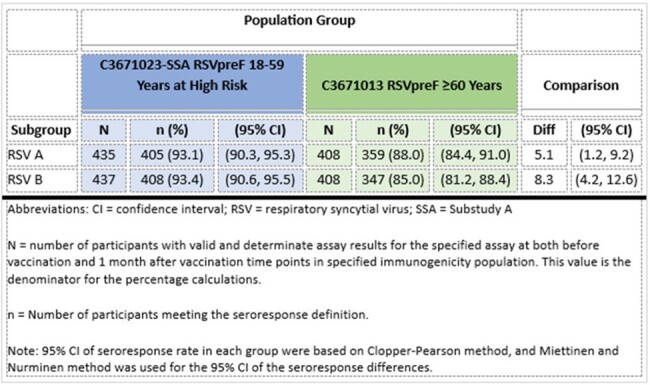
Comparison of RSV Neutralizing Titer Seroresponse Rates 1 Month After Vaccination with RSVpreF- 18 Through 59 Years at High Risk (C3671023) versus 60 Years and Older (C3671013)

**Conclusion:**

RSVpreF was well-tolerated with no safety concerns and demonstrated immunobridging to efficacy in adults 18-59 years of age at high risk of severe RSV disease compared to adults age ≥ 60. Results support the use of RSVpreF among adults 18-59 years of age with high-risk conditions to prevent RSV LRTI.

**Figure 1:**
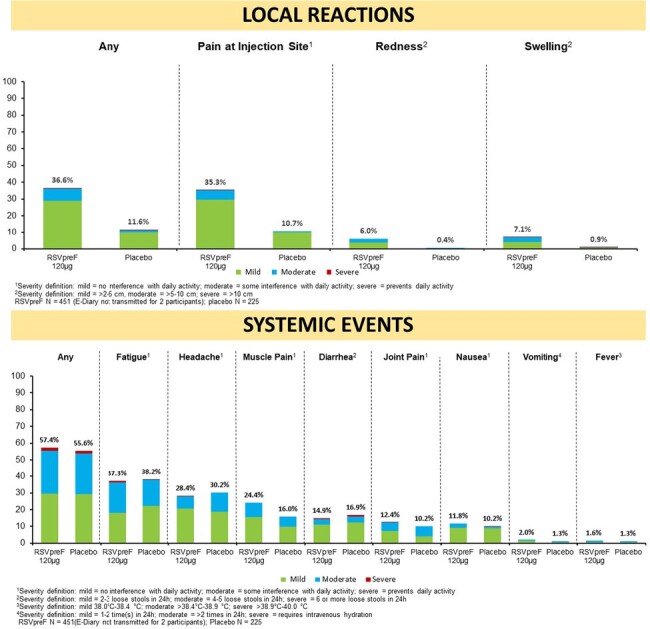
Local Reactions and Systemic Events Reported, By Maximum Severity, Within 7 Days After Vaccination with RSVpreF

**Disclosures:**

**William Towner, MD**, GSK: Grant/Research Support|Janssen: Grant/Research Support|Moderna: Grant/Research Support|Pfizer: Grant/Research Support **Elliot N. DeHaan, MD**, Pfizer, Inc.: Salaried employee|Pfizer, Inc.: Stocks/Bonds (Public Company) **Qin Jiang, PhD**, Pfizer: Salary|Pfizer: Stocks/Bonds (Public Company) **Wen Li, PhD**, Pfizer: stipend for my employment|Pfizer: Stocks/Bonds (Public Company) **Farah Rahman, DO**, Pfizer: Employee|Pfizer: Stocks/Bonds (Public Company) **Michael Patton, B.Sc.**, Pfizer: Employee|Pfizer: Stocks/Bonds (Public Company) **Hayley Wyper, BBiomedSc**, Pfizer: Stocks/Bonds (Public Company) **Maria Maddalena Lino, PhD**, Pfizer: Stocks/Bonds (Private Company) **Uzma N. Sarwar, MD**, Pfizer: Employee|Pfizer: Stocks/Bonds (Public Company) **Elena Kalinina, PhD**, Pfizer, Inc.: Salary|Pfizer, Inc.: Stocks/Bonds (Public Company) **David Cooper, PhD**, Pfizer, Inc.: Employee|Pfizer, Inc.: Stocks/Bonds (Public Company) **Kena A. Swanson, Ph.D.**, Pfizer: Employee of Pfizer|Pfizer: Stocks/Bonds (Public Company) **Annaliesa S. Anderson, PhD**, Pfizer, Inc.: Employee|Pfizer, Inc.: Stocks/Bonds (Public Company) **Alejandra C. Gurtman, M.D.**, Pfizer, Inc.: Employee|Pfizer, Inc.: Stocks/Bonds (Public Company) **Iona Munjal, MD**, Pfizer: Salaried employee|Pfizer: Stocks/Bonds (Public Company)

